# Design and Characterization of an Asynchronous Fixed Priority Tree Arbiter for SPAD Array Readout

**DOI:** 10.3390/s21123949

**Published:** 2021-06-08

**Authors:** Enagnon Aguénounon, Safa Razavinejad, Jean-Baptiste Schell, Mohammadreza Dolatpoor Lakeh, Wassim Khaddour, Foudil Dadouche, Jean-Baptiste Kammerer, Laurent Fesquet, Wilfried Uhring

**Affiliations:** 1 ICube Research Institute, University of Strasbourg, 23 Rue du Loess, CEDEX, 67037 Strasbourg, France; faguenounon@unistra.fr (E.A.); jbschell@unistra.fr (J.-B.S.); dolatpoorlakeh@unistra.fr (M.D.L.); wkhaddour@unistra.fr (W.K.); dadouche@unistra.fr (F.D.); jb.kammerer@unistra.fr (J.-B.K.); 2 Electronics Laboratory, Faculty of Engineering, University of Guilan, Khalij Fars Highway, Rasht 4199613776, Iran; safa.razavinejad@gmail.com; 3 TIMA, Grenoble INP, CNRS, University of Grenoble Alpes, 46 Avenue Félix Viallet, 38000 Grenoble, France; laurent.fesquet@univ-grenoble-alpes.fr

**Keywords:** SPAD, asynchronous logic, readout, micropipeline, fixed priority arbiter

## Abstract

The usage of single-photon avalanche diode arrays is becoming increasingly common in various domains such as medical imaging, automotive vision systems, and optical communications. Nowadays, thanks to the development of microelectronics technologies, the SPAD arrays designed for these applications has been drastically well-facilitated, allowing for the manufacturing of large matrices. However, there are growing challenges for the design of readout circuits with the needs of reducing their energy consumption (linked to the usage cost) and data rate. Indeed, the design of the readout circuit for the SPAD array is generally based on synchronous logic; the latter requires synchronization that may increase the dead time of the SPADs and clock trees management that are known to increase power consumption. With these limitations, the long-neglected asynchronous (clockless) logic proved to be a better alternative because of its ability to operate without a clock. In this paper, we presented the design of a 16-to-1 fixed-priority tree arbiter readout circuit for a SPAD array based on asynchronous logic principles. The design of this circuit was explained in detail and supported by simulation results. The manufactured chip was tested, and the experimental results showed that it is possible to record up to 333 million events per second; no reading errors were detected during the data extraction test.

## 1. Introduction

The single-photon avalanche diode (SPAD) is a photodetector component that is used for its ability to detect a single photon. It is commonly used in systems that measure sub-nanosecond time intervals [1,2,3,4]. In recent decades, they have been increasingly used as imaging array sensors [2,5] for various applications such as automotive advanced driver-assistance systems [6,7], biophotonics [3,8], and telecom transmission [9,10], among others.

The design of such sensors requires taking into account several aspects [3,4], such as the structure of the SPAD itself, the physical technology of the components, and the associated electronic circuits. The last point ranges from simple detection and quenching systems to more sophisticated ones incorporating photon counting, or integrating the ability to measure temporal resolution [11,12] and whether they integrate memory or not. These electronics can be built as close as possible to the SPAD cell or shifted to the end of the SPAD array. Regardless of the chosen options, the architecture of the SPAD-based sensor must be carefully thought out according to the application’s aim. Furthermore, integrating a readout system or/and a processing unit into the sensor is another important point to consider when designing such a sensor [13]. Indeed, integrating such systems has a direct influence on the amount of data that can be extracted from the sensor and the timing aperture.

A readout circuit for the SPAD array is of particular importance for real-time implementations. In general, existing readout systems can be grouped into two categories: sequential readout circuits and event-driven ones. The first category is based on synchronous logic and refers to the serial reading of each pixel of the matrix or one pixel column at a time. They have the advantage of being simpler to implement but do not allow for a fine chronological extraction of the data because of the need of synchronization to the clock; moreover, the use of clock trees makes the system more energy consuming [3]. In event-driven readout, however, a pixel is read only when an event is produced. By means of electronic circuits using asynchronous logic, the pixel output is applied to the controller, which starts its reading procedure. It is common for these systems to use a shared data bus with separate addressing lines. Such systems are more complicated to achieve but offer the advantage of reducing temporal aperture and energy consumption. A well-known system is the address-event representation (AER) readout method, which makes it possible to identify the pixel at which an event occurs as quickly as possible [14,15,16,17].

On event-driven readout systems, the release of the pixel so that it can perform a new measurement, and therefore the reduction of the temporal aperture, depends on the reaction time and read time of the controller. Indeed, photon detection being a purely asynchronous phenomenon, it is mandatory to have the ability to read and store this information immediately or at least release the SPAD as quickly as possible so that it can make a new measurement.

In this article, we proposed a readout circuit for a SPAD array-based imaging system. The studied circuit was inspired by the Network on Chip technology [18]. The principal aim of this work was to use a priority arbiter based on asynchronous logic to maintain the benefits of the event-driven method. The arbiter we proposed should handle both the address and the pixel data which leads to the release of the pixel as early as possible. Asynchronous reading of the data produced by the SPADs offered the advantage of reading without a clock tree, synchronization of clocks, and speed limitations inherent in synchronous logic and reduced the overall circuit power consumption. The structure we proposed in this work should allow reading from any type of SPAD pixel and SPAD array architecture (1D or 2D). The FPA circuit that we proposed aimed to transmit the data generated by the SPAD pixel. The data can be a timestamp of a Time Digital Converter (TDC), the intensity of the pixel, or just the hot pixel address, for instance.

The design and the characterization of this system are presented as follows. Section 2 describes our idea of the readout system based on the priority arbiter for SPAD imaging circuits. Section 3 presents an asynchronous logic principle needed for understanding the system operation. The design of specific asynchronous components required for the implementation of the studied system is reported in Section 4. In Section 5, we describe the priority arbiter we designed, starting with its structure, then discussing simulation results, and ending with its physical implementation. Section 6 shows the experimental validation we performed on the chip and discusses the advantages and the current limitations of the proposed method. Finally, a conclusion of this work is given in Section 7.

## 2. Asynchronous Priority-Based Arbiter Readout System

Priority arbitration involves the resolution of two or more competing signals requesting a shared resource from blocks running concurrently, such as SPAD pixels. Several arbitration methods are proposed in the literature to solve the problem of accessing resources for many applications [19,20]. In our case, that resource could have been a final readout system or processing unit. Priority arbiters can be classified according to the characteristics of their corresponding hardware implementation.

In a synchronous logic design using a clock signal, each request can be examined and one of them granted as the winner depending on the priority state machine. Nevertheless, a large array of SPAD can generate a high data rate that imposes the use of a high clock frequency, which considerably increases the power consumption.

In an asynchronous logic design, however, since there is no clock signal, the design must be able to handle a request signal at any time, and the grant must be guaranteed to be clean and safe, regardless of the signal arrival times. Such event-driven arbitration structures come with a minimal electrical activity that is proportional to access rates and without clock tree distribution issues [21]. Therefore, we chose asynchronous logic for the design of our priority arbiter.

Priority arbiters can be classified into two categories: fixed-priority arbiters (FPA) and dynamic ones. In fixed-based systems, the FPA has to choose between input requests with predefined hardware-coded priority values. In other words, the FPA compares the priority of an event that occurs in one of the FPA’s inputs with all the other events happening or that have already happened and then sends each event to the output based on its predefined priority until the last of them has been processed. In contrast to FPA, dynamic priority arbiters (DPA) are able to dynamically change input priority. Consequently, DPAs have lower speeds and are more complex to design and implement.

In this work, in order to avoid the speed limitation inherent to DPAs, we decided to design and use a fixed-priority tree which is presented in the following sections [22].

**Figure 1:**
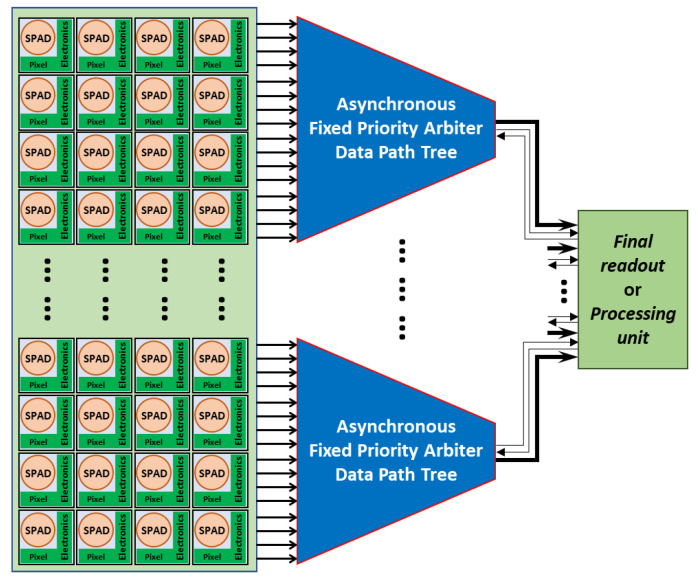
Synoptic of a possible SPAD array readout system using the proposed asynchronous FPA tree. The SPAD pixels generate some data that are extracted from the array thanks to the FPA.

To illustrate the concept, Figure 1 illustrates a block diagram of a SPAD-based imaging sensor integrating an asynchronous FPA data path tree before the final readout or processing unit. In the case of a SPAD sensor array, the data can be a single bit indicating that a pixel has detected a photon, or the value delivered by a time to digital converter (TDC), or any kind of timestamp or intensity-related value transmitted by the pixel. To understand the operation mode of such a system, the asynchronous logic principles are detailed in the next section.

**Figure 2:**
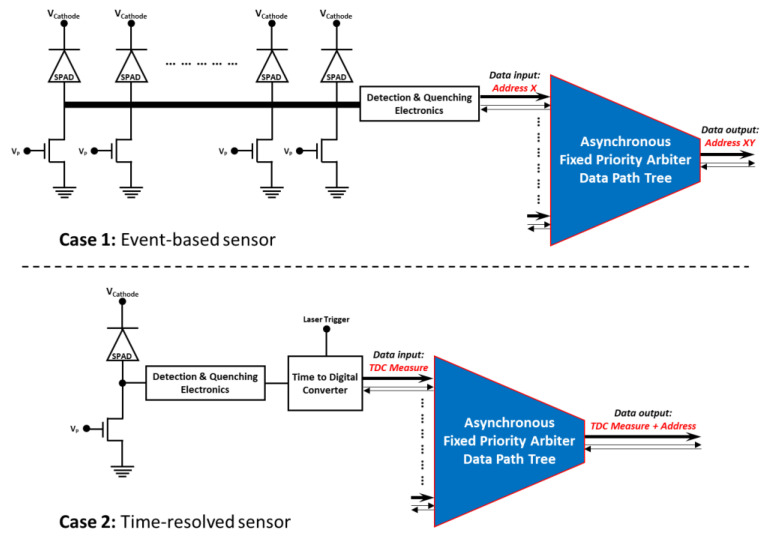
Application examples of the FPA tree implementation within SPAD-based systems. Case 1: an event-based sensor for low light imaging. Case 2: a time-correlated single-photon counting image sensor.

To highlight how such a circuit can be implemented with a SPAD sensor, two scenarios are proposed and presented in Figure 2. In each case, the SPADs and their associated electronics are connected to the input of the FPA tree, but a different type of data can be generated by the SPAD pixel. In case 1, the SPAD array is used as an event-based sensor [23] for low-light level imaging. The SPAD pixel detecting a photon provides, at the input of the FPA tree, the column address X of the activated SPAD within the row. The FPA tree extracts the data presented at it input and encodes the row address Y. As a consequence, the system identifies the SPAD that detected a photon and provides its addresses X and Y at its output. In this case, the fast data extraction allows for the easy generation of a sequence of images at a classic video rate.

In case 2, the SPAD is used to provide the temporal information on detected events in regards of a laser trigger, i.e., a laser pulse, thanks to a time to digital converter. In this way, the system can be seen as a parallelized time-correlated photon counting system (TCSPC) [12] with a few tens of temporal resolution. Additionally, the row and column addresses X and Y can also be added with the same scheme as in case 1 in order to have a time-resolved image sensor [24]. In this case, the fast data extraction allows for a high photon count rate TCSPC.

## 3. Asynchronous Logic Principle

Asynchronous logic constitutes a clockless circuit. Since there is no clock in the circuit, techniques and protocols were developed to ensure good data transfer between the different components and stages of the asynchronous circuit. In this section, we introduced the essential ones for our fixed-priority arbiter tree design. The latter covers the micropipeline circuit, the handshake protocols, and the data encoding and the fixed-priority arbiter basic operation aspects.

### 3.1. Micropipeline Circuit

**Figure 3:**
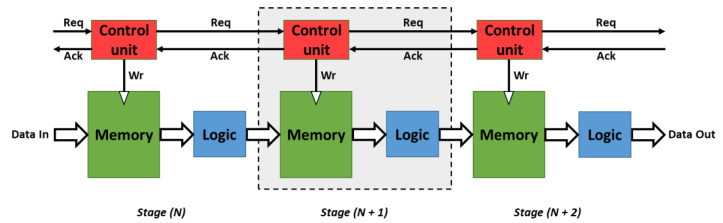
Basic micropipeline circuit. Each stage consists of a control unit and a process unit. The communication protocol between two stages is ensured by request and acknowledge signals.

Pipelining is a fundamental technique to increase concurrency and boost throughput in high-performance digital systems. In an asynchronous system, the word micropipeline was introduced by Ivan E. Sutherland [25], referring to a particularly simple circuitry form of event-driven elastic pipelines with or one without internal processing. Figure 3 shows a basic micropipeline structure [26] in which the data moves between the pipeline stages according to an established request-acknowledge mechanism. Typically, after data moves through an individual stage’s memory (*N*), the corresponding controller unit (*N*) requests, by means of its request signal (Req), the next control input (*N* + 1) to store the data in its memory (*N* + 1). Once the data moves through the next stage’s memory (*N* + 2) to be stored again, a transition on the acknowledge signal (Ack) of that stage (*N* + 2) makes the current stage’s memory (*N* + 1) available, thus completing an entire cycle. Briefly, a stage (*N*) cannot consider a new request signal (data) if it does not receive the acknowledge signal from its previous transfer. The interaction of neighboring stages is coordinated by using the handshaking protocol and data encoding schemes wherein the request signal is going forward and the acknowledge signal is going backward.

### 3.2. Handshake Protocols and Data Encoding

Handshaking protocol and data encoding techniques are largely described in the literature [25,26]. In this section, we only provided a brief recall of their principles.

#### 3.2.1. Handshaking Protocols

There are two protocols frequently used to manage data transfer between a sender unit and a receiver one: (i) non-return-to-zero handshake protocol (2-phase handshaking) and (ii) return-to-zero handshake protocol (4-phase handshaking).

**Figure 4:**
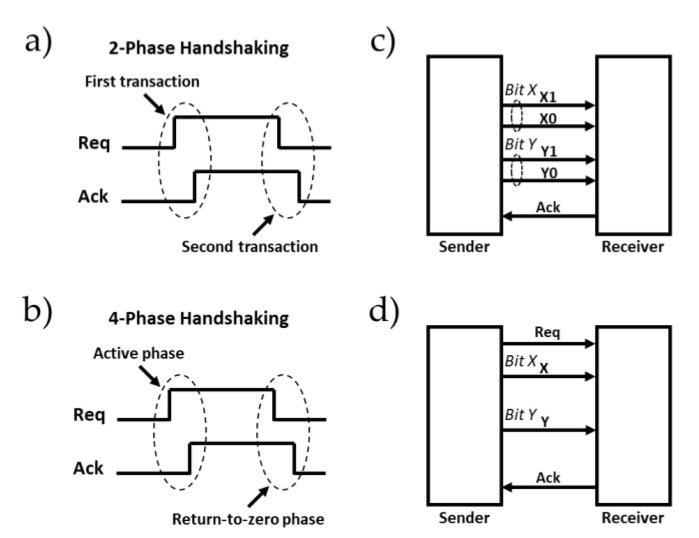
(**a**) Non-return-to-zero handshake protocol. (**b**) Return-to-zero handshake protocol. (**c**) Dual-rail data encoding. (**d**) Single-rail data encoding [26].

In 2-phase handshaking (Figure 4a), a single toggle (low-to-high or high-to-low) on the Req signal followed by a toggle-on Ack signal completes one transaction. The main advantage of this technique is its high throughput, but its hardware design is more complex.

In 4-phase handshaking, illustrated by Figure 4b, the hardware design is easier. The functioning principle involves four steps defined by the control signals Req and Ack. Initially, Req and Ack are both low, and then the Req signal is asserted which causes, in turn, the assertion of the Ack signal. To complete a full transaction, this step is followed by returning the Req as well as the Ack signals to their initial state.

In this paper, we used a 4-phase handshaking protocol due to its simple design. The only difference in our protocol compared with that shown in Figure 4b is that its Ack signal was initially set to the high-level state.

#### 3.2.2. Data Encoding Schemes

There are two data encoding categories for asynchronous communication: (i) the delay-insensitive data encoding method, and (ii) the single-rail bundled-data method. In delay-insensitive encoding, there is no dedicated wire for the Req signal. Instead, the data bus is encoded, and each stage integrates a combinatorial circuit which checks the input-data code and generates a data valid signal. This signal is used as the request signal. Dual-rail code, illustrated in Figure 4c, or other m-of-n codes can be used [27]. In single-rail bundled-data encoding schemes (Figure 4d), a natural data bus is used. In this paper, single-rail bundled-data encoding was used due to its higher coding efficiency and low area occupancy.

### 3.3. Fixed Priority Arbiter

**Figure 5:**
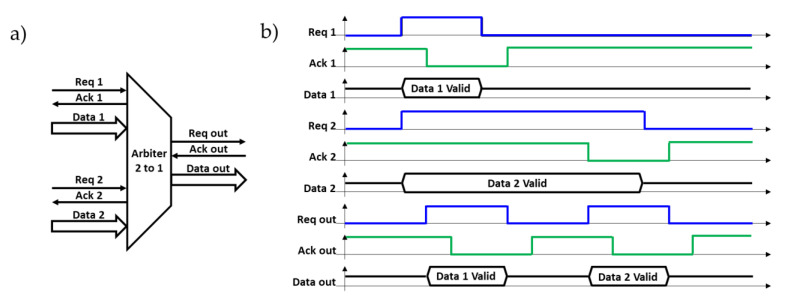
(**a**) Fixed-priority two-to-one arbiter managing to extract the data generated by two different units connected at its input. (**b**) Functioning principle illustration of the two-to-one arbiter when the two input data are presented at the same time. The first data are extracted followed by the second.

The basic FPA module is a 2-input arbiter which is shown in Figure 5a. If there is only one request in the inputs, then the FPA sends the related data to the output. This means that the request signal and the data propagate through the arbiter and the sending unit is acknowledged after the data transfer is achieved. However, if both requests activate simultaneously, the arbiter should allow just one of the input signals to be conveyed to the output according to its priority. In the case of two input requests at the same time, the proposed FPA in our work always allowed the first channel (**Req 1**, **Ack 1**, **Data 1**). This is shown in Figure 5b. After granting the first input signal, the second input can be granted as soon as the next block acknowledges the current block with the **Ack out** signal. When **Req out** is sent to the next component, the previous blocks (1 and 2) are acknowledged with **Ack 1** and **Ack 2** signals, respectively.

In more detail, the two-sender units of this example request and present valid data on the data bus simultaneously. The data remain stable as long as the request is active at the high level. **Req 1** has the highest priority, **Ack 1**, for which we have chosen to set the zero level to the high state in our design, goes to the low state, and, after a delay, **Req 1** returns to the low state followed by the return of **Ack 1** to the high state. **Req 2** has the lowest priority, and its level remains high at the time that the valid **Data 1** is transmitted to the next stage by a cycle (**Req out**, **Ack out**). Finally, once unoccupied, the FPA can transmit the valid **Data 2**.

## 4. Design of the Studied System

The implementation of asynchronous systems requires the design of specific components. More particularly, in our system, to implement the fixed-priority tree, we used 2-input static Muller C-elements. This section covers the design of the Muller C-element as well as the full design of our 2-input fixed-priority arbiter.

### 4.1. Muller C-Element (C-Muller)

**Figure 6:**
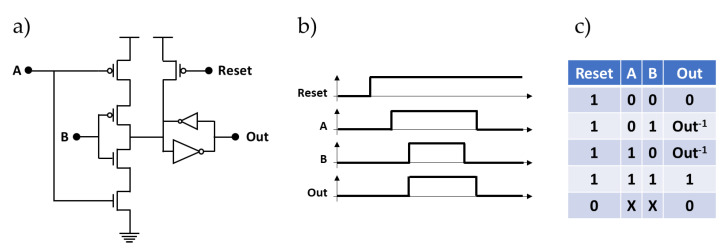
(**a**) Transistor-level design of the muller gate. (**b**) Timing diagram. (**c**) Truth table.

A Muller C-element is a commonly used component in the design of asynchronous circuits. It is used for joining signal transitions (events) or completion time detection. Figure 6a illustrates a static implementation of the Muller C-element using complementary metal oxide semiconductor (CMOS) technology. This element uses three PMOS transistors, two NMOS transistors, and two inverters (a normal one and a weak one). When both inputs **A** and **B** are 0, the pull-up network changes the output state **Out** of the C-element to 0 through the normal inverter. When both inputs **A** and **B** are 1, the pull-down network changes the output state **Out** of the C-element to 1 through the normal inverter. In the other cases, the output **Out** of the C-element is not connected to either **Vdd** or **Gnd**, and the weak inverter (shown here with a smaller symbol) performs with the normal one an internal storage to retain the previous state on the output **Out**. In addition to the two inputs **A** and **B**, we added an active low **Reset** input that allows the assertion of the output **Out** to 0. These different operation modes are summarized by the timing diagram and the truth table respectively shown in Figure 6b,c.

### 4.2. Fixed Priority Arbiter Unit

**Figure 7:**
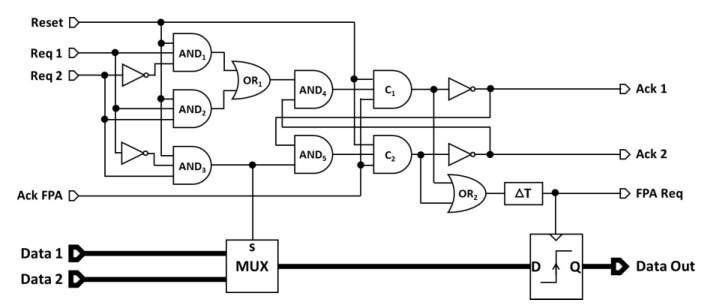
The proposed two-to-one FPA schematic. The upper part is the control unit that manages the priority and the Request/Acknowledge protocol. The lower part is the process unit that selects the good data to be stored in the flip-flop register according to the requested input Req1 or Req2.

The principle of the 2-input fixed-priority arbiter architecture is schematically presented in Figure 7. This architecture consists of two parts: a control unit and a data path (for memory storage).

**Control unit:** The control unit has, at its entrance, three AND gates which serve to determine which request signal will be granted. If **Req 1** is active and *t* **Req 2** is not, the output of the gate **AND_1_** is set to 1; If **Req 1** and **Req 2** happen simultaneously, the output of the gate **AND_2_** is set to 1. These two configurations are grouped in one by the gate **OR_1_**; its output is then set to 1, meaning that **Req 1** is granted. When **Req 2** is active and **Req 1** is not, the output of the gate **AND_3_** is set to 1, meaning that **Req 2** is granted. This gate output is also guiding related data to the output by setting the selection pin of the two-to-one multiplexer. The control unit also has a **Reset** input, which acts on the three AND gates and on the two Muller C-element to reset the FPA.

The two trios (**AND_4_**, **C_1_** and inverter) and (**AND_5_**, **C_2_** and inverter) execute the 4-phase handshaking protocol with the granted access request, and the mutual exclusion ensures only one sequence at a time. Assuming that the outputs **Ack 1** and **Ack 2** are initially set to 1 (i.e., the unit is free and no transfer is in progress), and the input **Ack FPA** is set to 1 (i.e., the next stage is also free), and **Reset** is set to 1, when the **Req 1** is granted, the output of **AND_4_** changes to 1, the output of the Muller C-element becomes 1, and the output of **Ack 1** changes to 0. This signal is fed back for mutual exclusion on the competing trio and forces the **Ack 2** output to be maintained at 1. The output signals of the two Muller C-elements pass through the **OR_2_** gate and are then delayed by (**∆T**) to generate the **FPA Req** signal that triggers a request to the next stage. Furthermore, the rising edge of this signal allows for the storing of the data in the memory. Once the **Ack 1** signal is received by the previous stage, the **Req 1** becomes 0 and thus the output of the **AND_4_** gate. As soon as the next stage changes the **Ack FPA** signal to 0, the output of the Muller C-element changes to 0 and **Ack 1** returns to 1 again, ready for a new cycle.

**Data path:** On the data path, a two-to-one multiplexer was used to choose one of the two data inputs related to the request signals. The selection input of this multiplexer is connected to the output of the **AND_3_** gate. When the latter is set to 0 (**Req 1** is granted), **Data 1** is transmitted to the storage unit. In the other case, when **Req 2** is granted, **Data 2** will be selected. The **FPA Req** signal that comes from the output of the **OR_2_** gate delayed by (**∆T**) acts as a local clock for the memory unit. A rising edge of this signal stores the selected data in the memory and makes this data available on the output **Data Out**. To ensure the correct operation of the data path, the data should be stable at the memory input before the **FPA Req** signal transition. Furthermore, once the data enter a stage, they must be securely stored before new data are sent by the previous stage as explained in Section 3.1. Theses constraints were satisfied by adding a delay module (**∆T**) of 1 ns which ensures the local timing of the FPA.

## 5. VLSI Implementation of the Proposed 16 to 1 Fixed Priority Arbiter

### 5.1. Tree-Structure of the Proposed 16 to 1 FPA

**Figure 8:**
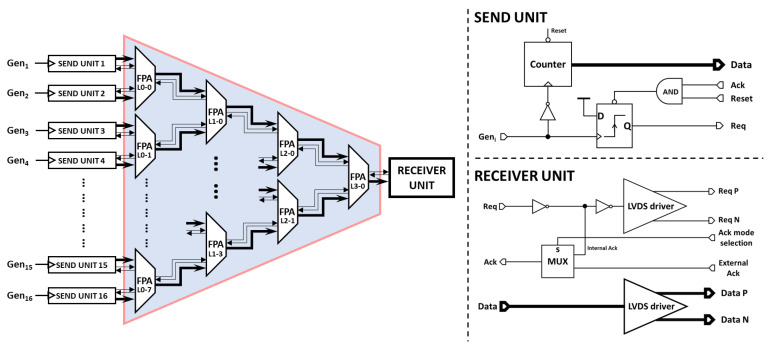
FPA 16-to-1 tree connected to 16 send units for testing purposes. The first column of the tree is named L0, the second one L1, and etc. The send units consist of a simple counter to generate well-controlled data and a flip flop is used to handle the asynchronous communication protocol. The receiver unit allows for the extraction of the data out of the chip thanks to its high-speed LVDS driver.

N-way arbiter structures have been widely discussed in the literature [28,29]. The N-way tree arbiter is one of them; it is typically made by cascading several 2-input arbiters which are used for each arbitration node on the tree. The schematic view of our proposed 16-way fixed-priority tree arbiter is shown in Figure 8. Each input client of this tree will be attached to a designated lowest-level FPA two-to-one unit. To be read out, a client must have the arbitration priority at each level until the top level. The last stage of the arbiter tree can be then connected to the readout circuit of an eventual processing unit.

In some applications, it is mandatory to know the location of the pixel. Obviously, the pixel itself can transmit its location in addition to its generated data. Then, the FPA tree transmits the data flow from the input node of the tree to its output node with a fixed data bus. The drawback of this technique is that wide data width memories are needed all along the data path, which consumes a large silicon area. Indeed, in order to minimize the used area of silicon, another addressing method was proposed. The main idea is explained as follows: at each two-to-one FPA node, an additional bit is added to the data to encode the origin of the request signal. This bit will be “0” if the origin is the **Req 1** and “1” if the origin is **Req 2**. This is created by adding the select signal of the multiplexer (the output signal of the **AND_3_** gate) as the least-significant bit (LSB) for the **MUX** output data bus. Each path from the input nodes can then be encoded with a specific address. This address encoding method requires a specific two-to-one FPA for each level of the arbitration tree, and the memory of each level should be 1 bit larger than the memory of the previous one.

### 5.2. Send Units

In order to test the designed FPA tree, 16 send units were built to feed the lowest-level two-to-one FPAs (L0-j, with j ∈ [0;7]) with 4-bit data (Figure 8). All the send units were similar, and each unit consisted of a 4-bit counter and included a request signal generation unit with asynchronous acknowledgement signal reception. These counters were synchronous and worked on the Gen_i_ falling edges, and the request signals were generated on the Gen_i_ rising edges. The 16 send units were fed by four different signals configured as follows: a common signal for units 1 to 8 (i.e., Gen_1_ to Gen_8_ are connected together), one for unit 9, one for unit 10, and finally one common signal for units 11 to 16 (i.e., Gen_11_ to Gen_16_ are connected together). This configuration made it possible to excite the send units differently: (i) the first 8 units together at the same time, (ii) the units 9 and 10 independently, and (iii) the last set of units (11 to 16) also at the same time.

At the output of the FPA tree, we have a receiver unit which outputs the data out of the chip for the highest-level two-to-one arbiter (L3-0) node to a bus of an 8-bit low-voltage differential signaling (LVDS) driver. The **FPA Req** signal of the last node is also available on an LVDS link. Finally, this unit integrates a multiplexer to operate in two acknowledgement modes (internal and external acknowledgement modes). In the internal mode, the multiplexer transmits the internal Ack signal generated by the chip itself. In the external mode, the external Ack generated by an FPGA is selected.

### 5.3. Simulation Results

**Figure 9:**
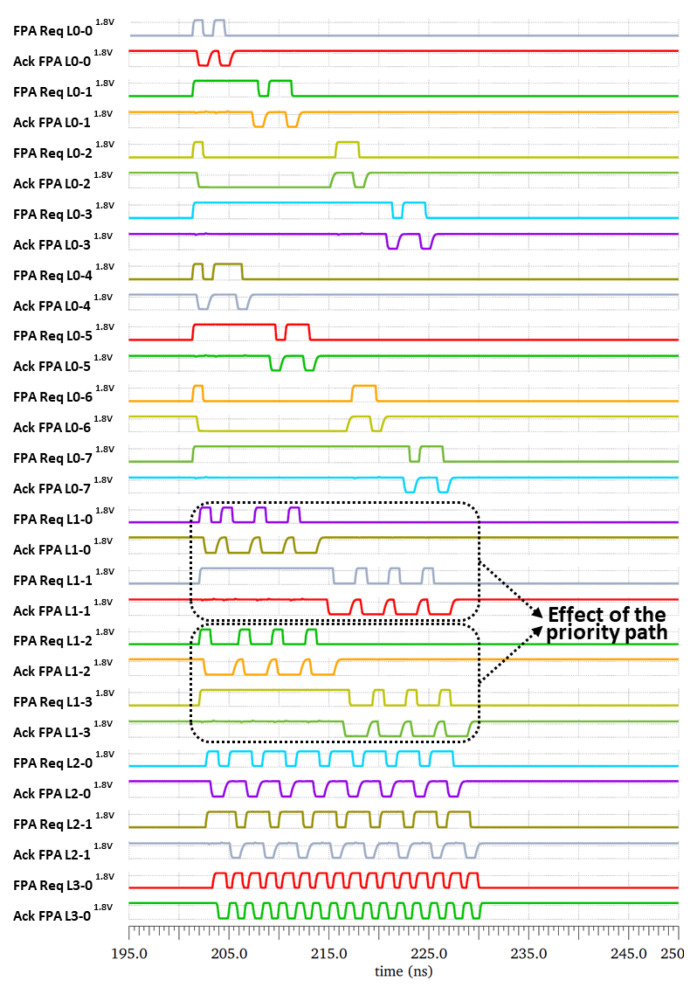
FPA tree Cadence simulation results. The effect of the priority path is highlighted with the output of the second row of the FPA the request signal L1-0 and L1-1. The L1-0 data are extracted first by the next L2-0 FPA unit.

The FPA tree was simulated using the “Cadence Virtuoso Analog Design Environment”. The results of the worst-possible case (when all the 16 send units requested simultaneously) are presented below. Figure 9 shows the output of all FPAs and demonstrates the functionality of the FPA tree as well as the priority of one branch of the tree over the other ones. The timing diagram showed that the FPA L0-0 sent two request pulses to the FPA L1-0 to transfer the data from the two send units 1 and 2. It also showed that the output of the last FPA L3-0 generated 16 request pulses indicating that the data of the 16 send units had been transferred to the receiver unit.

Two cases were highlighted by two dotted rectangles in order to illustrate the effect of the priority. In the first case, since FPA L1-0 had priority over FPA L1-1, and the FPA Req L1-1 signal (indicated in gray color) waited longer before being granted. This allowed the FPA L1-0 to prioritize the outputs of the FPA L0-0 and FPA L0-1. The same behavior was observed between FPA L1-2 and FPA L1-3.

**Figure 10:**
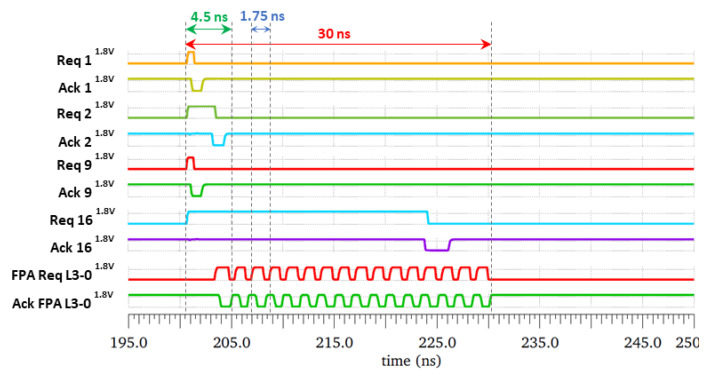
Best- and worst-case latencies simulation results in the case where all the 16 send units are triggered simultaneously. The highest priority datum, the Req1 signal, is extracted first with a latency of only 4.5 ns and the lowest priority datum, the Req16 signal, is extracted after all the other ones with a latency of 30 ns.

Figure 10 compares the time taken for a request to be considered and handled by the last FPA of the tree. Observing the latency of the request signal for some units (for example unit 1, 2, 9 and unit 16), the shortest latency was reported for Req 1 (or Req 9) and equal to 4.5 ns, and Req 16 had the worst latency which equaled to 30 ns. The simulation results also revealed that a full cycle on the last FPA of the tree took 1.75 ns.

### 5.4. Physical Implementation

**Figure 11:**
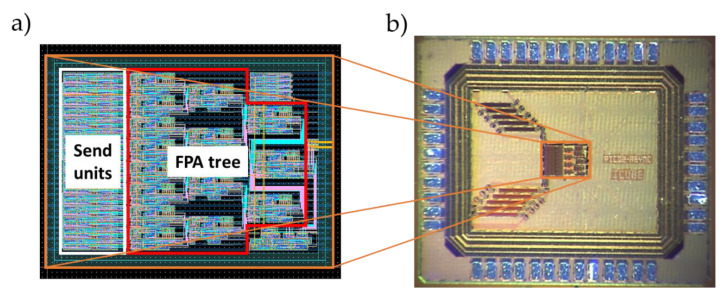
(**a**) FPA tree layout. (**b**) The full device front view by microphotography.

The circuit was designed with 180 nm CMOS process technology. Figure 11a illustrates the core of the FPA tree layout with the test purpose unit. The occupied silicon area of the FPA tree was 196 µm × 180 µm, 260 µm × 180 µm with the send units included. Figure 11b shows a microphotograph of the manufactured chip. The total size of the chip was 1785 µm × 1500 µm.

## 6. Experimental Environment, Results and Discussion

### 6.1. Experimental Setup

**Figure 12:**
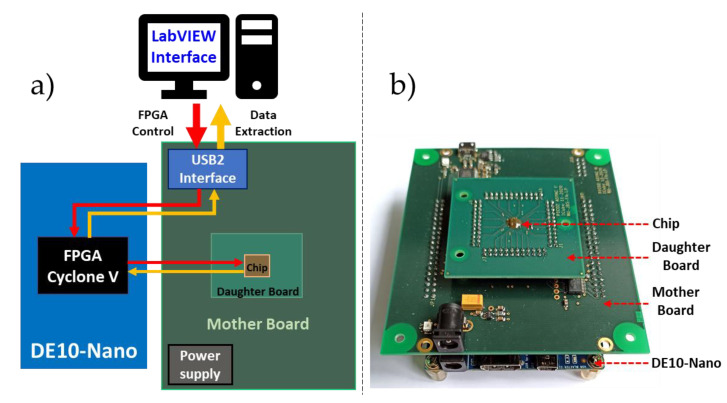
(**a**) Block diagram of the experimental setup which consists of a stack of three boards: a daughter board with the design CHIP, a motherboard which meanly provides the power supply, and the USB2 interface and a commercial FPGA design kit. (**b**) Picture of the experimental setup.

In order to characterize the prototype, a testing platform based on Field Programmable Gate-Array (FPGA) DE10NANO (Terasic Inc., Hsinchu City, Taiwan) board was developed as shown in Figure 12. The test chip was mounted on a daughter board plugged into a motherboard. The motherboard integrated (i) the power supply for the chip, (ii) circuits for the configuration of the chip (i.e., the bit stream sent by the FPGA), (iii) high-speed differential receivers to enable the FPGA to read the LVDS signals, and (iv) a USB2 controller for the communication with a custom LabVIEW interface. The LabVIEW interface controlled the system and read the output data. It configured the FPGA (a) to send the configuration bits to the chip, (b) to provide the excitation signals for the send units, and (c) to read the output data stream of the tree (when operating in external mode). The configuration bits stream activated the LVDS link and selected the acknowledgement mode. Finally, a 13 GHz wideband oscilloscope and a 6 GHz differential probe were used to measure the LVDS signals.

### 6.2. Results and Discussion

**Figure 13:**
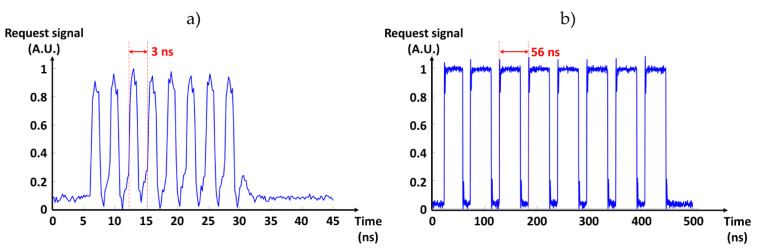
Last FPA request timing measurement. (**a**) Internal acknowledge mode with an output cycle of only 3 ns. (**b**) External acknowledge mode with an output cycle of 56 ns limited by the FPGA operating frequency.

The developed test bench was used for the experimental validation of the FPA tree by carrying out a set of experiments. In the first experiment (in the case of a simultaneous requests from the first eight send units in the internal acknowledgement mode) we measured the timing of the LVDS Req signal output of the last stage of the FPA tree using the differential probe. Figure 13a presents the obtained timing measurements and shows that it took 3 ns to complete an output cycle (i.e., 24 ns for the 8 cycles). The maximal data rate of the FPA tree was then estimated to 333 MHz. It is worth noting that the delay of the internal acknowledge signal was chosen to be 1 ns, which is a safe delay in the used technology to ensure the functionality of the device. A shorter delay (<1 ns) can be chosen to increase the total throughput.

The same experiment (eight simultaneous requests signals) was repeated in the external acknowledgement mode and the results are presented in Figure 13b. In this mode, the throughput was limited by the FPGA acknowledgment signal generation rate and a single cycle was completed in 56 ns. Hence, this mode was slower than the internal acknowledgement mode, but it allowed for the recording of the data by the FPGA.

**Figure 14:**
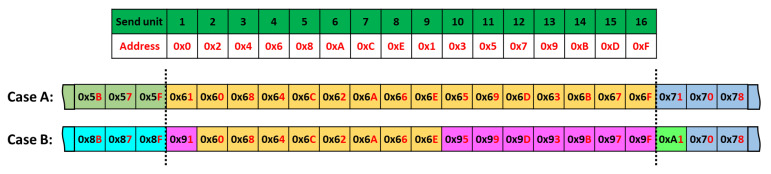
Readout experience data obtained in case A and in case B. The order in which the data are extracted from the sensor is related to the priority imposed by the FPA tree.

In the next experiment, we recorded the data readout at the output of the FPA tree for two different configurations: (case A) all the 16 units started simultaneously so that the counter value was the same for all of them, and (case B) units 1 to 8 and units 9 to 16 started with an offset, so they had different counter values. In both configurations, 187 million events were recorded. Figure 14 presents a section of the recorded data in a hexadecimal format that allows assessing the result in both cases with the arriving order of the data. The lower nibble stands for the send unit address (i.e., the related number of the send unit), and the upper nibble stands for the counter value of the send unit. In this figure, the link between the number of the units and their addresses is listed in the upper table. The addresses are also highlighted by the red color in the displayed frames (case A and case B). In case A, all the 16 recorded values were equal. In case B, the values received from the send unit 1 to 8 were different than the ones received from the send unit 9 to 16, as expected. In both cases, all the collected data were analyzed, and no transmission error was detected for the 187 million measured events.

In the last experiment, we measured the power consumption (volts, amperes) of the proposed FPA tree while all the LVDS drivers were disabled in two states: (1) the idle state wherein the global reset signal was activated, and (2) the fully loaded operating state where request signals were generated on the 16 sender units at a rate of 25 MHz (at this request rate, the FPA tree was fully loaded leading to the maximum event rate of 333 million events per second). Comparing the power consumption for the two states indicated an estimated average power consumption of 1.8 milliwatts (1.8 V, 1 mA) for the FPA tree which can be reported as 5.4 picojoules per complete event readout (i.e., the power needed to transmit data from a send unit up to the last FPA stage).

These experiments allowed us to validate and demonstrate the good functionality of the proposed FPA tree structure. However, we have noted some observations and some points that can be improved in this design. Fixed-priority arbiters always select the request input port of the highest priority, and the input ports with lower priority may rarely be granted when requiring conflicts happen. To ensure that, it is necessary to be very precise during the layout design. In our design, we saw that some Req signals which should have had a lower priority came out first, but this issue did not influence the logic behind our project. For example, the send unit 9 came first in both the simulation and the experimental measurement. Practically, it is very improbable that all the SPADs of an array are triggered at the same instant. However, even if that happens, the integrity of data will still be valid, but only the order in which the data are collected will be different.

Overall, in this work we demonstrated that asynchronous 16-to-1 FPA trees can be used to design a reliable readout circuit with low power consumption, high supported event rate (up to 333 Million events per second), and low cost (small silicon area). Based on the obtained results, the designed circuit could be extended to support ultra-fast phenomena measurements using larger SPAD matrix sensors (>16 SPADs). To achieve this, several FPA trees should be implemented to run in parallel. For example, with a 16 × 4 SPAD matrix, four 16-to-1 FPA trees would be required in addition to a final readout unit or a processing unit. We could be able to measure up to 1.3 giga-events per second with a worst latency of 48 ns (for the last request signal in the case in which all the SPADs of the matrix are activated at the same time). Such a circuit would require a final readout unit which should be capable of handling a high-speed data rate (up to 15.6 Gbps for a 12-bit data path [8-bit SPAD + 4-bit address] and 26 Gbps for a 20-bit data path [16-bit SPAD + 4-bit address]) such as CoaXPress technology. Another solution could be the integration of the processing unit in the sensor to reduce the amount of data.

This circuit is also intrinsically well suited to handle asynchronous and Poissonian processes such as that of a SPAD array. Indeed, when a burst of photons is detected, the data will be immediately collected and stored inside the FPA tree. Then, the send units (i.e., the pixels electronic) will be released as soon as possible. The FPA tree itself acts as a buffer memory that allows for handling the inherent peak of activity at its input due to the quantic behavior of the SPAD array. As reported in [30], the use of a memory buffer such as a FIFO enables the SPAD array sensors to detect and read out a photon rate equal to the system’s maximum readout data rate with a high efficiency of more than 90% (i.e., less than 10% of missed photons), whereas the efficiency of a bufferless system is only 50% in the same conditions.

In summary, there is no doubt for us that such a structure will pave the way for the development of fully asynchronous photon counting systems operating at a high photon rate with a low latency and a low power consumption.

## 7. Conclusions

A new design for asynchronous FPA was presented and used to build an event-driven FPA 16-to-1 data path tree for SPAD-array readout. This design was implemented in a 180 nm CMOS technology. The good functioning of the design circuit was validated in simulations and by performing experimental measurements. In particular, the performed experiments showed that, with such a readout circuit, it is possible to read up to 333 million events per second. Furthermore, this design is simple to implement and requires no clock distribution tree. It is also compact, occupies a low silicon area, and benefits from low power consumption. In future work, the 16-to-1 FPA tree should be integrated in the readout circuit for our SPAD-array-based sensor that will be used in the development of our future SPAD-based streak camera.

## Data Availability

Not applicable.
